# *Akkermansia muciniphila*, which is enriched in the gut microbiota by metformin, improves cognitive function in aged mice by reducing the proinflammatory cytokine interleukin-6

**DOI:** 10.1186/s40168-023-01567-1

**Published:** 2023-05-30

**Authors:** Xiaoqi Zhu, Junyan Shen, Shengyu Feng, Ce Huang, Hao Wang, Fengjiao Huo, Hailiang Liu

**Affiliations:** 1 Institute for Regenerative Medicine, Shanghai East Hospital, Tongji University School of Medicine, Shanghai, 200123 China; 2 Key Laboratory of Xinjiang Phytomedicine Resource and Utilization of Ministry of Education, College of Life Sciences, Shihezi University, Shihezi, 832003 China; 3 Institute of Advanced Biotechnology, Southern University of Science and Technology, Shenzhen, 518055 China

**Keywords:** *A. muciniphila*, Metformin, Gut microbiota, Cognitive function, Inflammation, IL-6

## Abstract

**Background:**

Metformin, a type 2 diabetes treatment, improves the cognitive function of aged mice; however, whether the protective effects of metformin on cognitive function in aged mice are associated with the gut microbiome is poorly understood. Although some studies suggest that the gut microbe composition influences cognitive function and that manipulating the gut microbiota might protect against age-related cognitive dysfunction, there is no direct evidence to validate that the gut microbiota mediates the effect of metformin on cognitive improvement.

**Results:**

In this study, we show that the gut microbiota is altered by metformin, which is necessary for protection against ageing-associated cognitive function declines in aged mice. Mice treated with antibiotics did not exhibit metformin-mediated cognitive function protection. Moreover, treatment with *Akkermansia muciniphila,* which is enriched by metformin, improved cognitive function in aged mice. Mechanistically, *A. muciniphila* decreased pro-inflammatory-associated pathways, particularly that of the pro-inflammatory cytokine interleukin (IL)-6, in both the peripheral blood and hippocampal profiles, which was correlated with cognitive function improvement. An IL-6 antibody protected cognitive function, and an IL-6 recombinant protein abolished the protective effect of *A. muciniphila* on cognitive function in aged mice.

**Conclusion:**

This study reveals that *A. muciniphila*, which is mediated in the gut microbiota by metformin, modulates inflammation-related pathways in the host and improves cognitive function in aged mice by reducing the pro-inflammatory cytokine IL-6.

Video Abstract

**Graphical Abstract:**

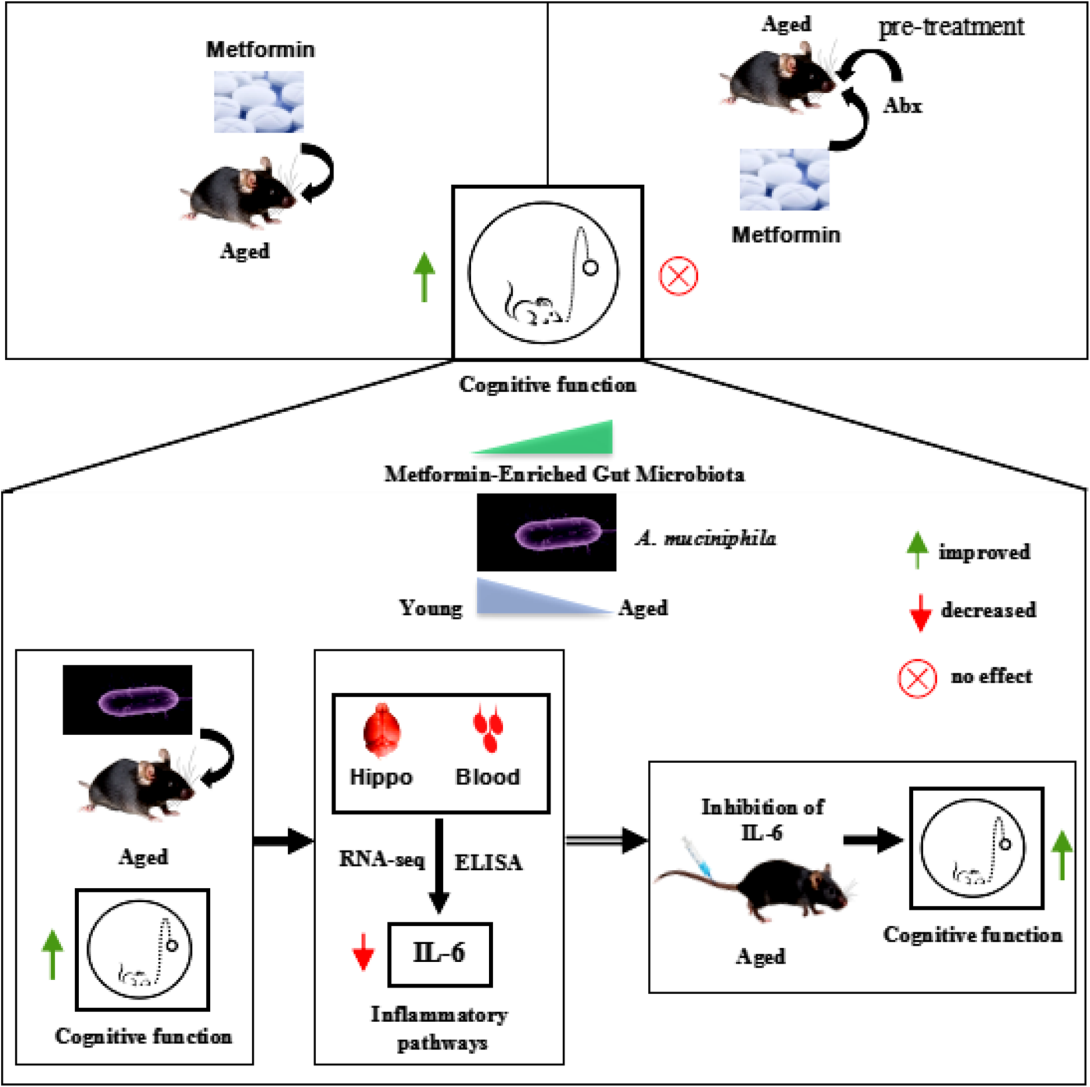

**Supplementary Information:**

The online version contains supplementary material available at 10.1186/s40168-023-01567-1.

## Background

Metformin is a first-line treatment for type 2 diabetes. However, in addition to its value for treating type 2 diabetes, it is increasingly applied for the treatment of other disorders, including autism spectrum disorder, Alzheimer’s disease, metabolic syndrome, cancer, and ageing [1–3]. Unexpectedly, the survival of type 2 diabetes patients treated with metformin was prolonged compared with that of matched controls [3]. Recently, researchers have shown that metformin significantly extends the life span of nematodes, rodents, and primates [3–7], but the mechanism underlying its anti-ageing effects remains unclear.

Recent studies of animals and humans have indicated that the gut microbiome is a major site of action of metformin. Metformin induces a profound shift in the composition of the gut microbiota community, which might contribute to the anti-diabetic effect of metformin [8–11]. The effects of metformin on host physiological functions are affected by its interactions with symbiotic microbiota in an evolutionarily conserved manner, from nematodes to more evolved organisms [8, 9, 12, 13]. The anti-ageing effects of metformin are also related to changes in the microbiota, which have been investigated in nematodes [12, 14, 15]. Metformin improved the cognitive function of aged mice in our previous study [7]. However, whether the protective effects of metformin on cognitive function in aged mice are associated with the gut microbiome is poorly understood.

Gut microbes influence neuro-regeneration and behaviour via the gut-brain axis. Furthermore, the gut-brain axis and gut microbiota play critical roles in orchestrating brain development and behaviour [16–21], and the immune system is emerging as an important regulator of these interactions [16]. The gut microbiota modulates the maturation and function of tissue-resident immune cells in the central nervous system (CNS) [19]. The gut microbiota also influences the activation of peripheral immune cells, which regulate responses to neuroinflammation and brain injury, autoimmunity, and neurogenesis [22, 23]. Accordingly, both the gut microbiota and immune system participate in the etiopathogenesis or manifestation of neurodevelopmental, psychiatric, and neurodegenerative diseases, such as autism spectrum disorder, depression, and Alzheimer’s disease [16, 24–26]. The gut microbiota has also been increasingly associated with changes in factors relevant to neurotransmission, including neurotransmitter signalling, synaptic protein expression, long-term potentiation, and myelination, as well as a variety of complex host behaviours, including stress-induced, social, and cognitive behaviours [16, 27]. These studies suggest that the gut microbe composition influences cognitive function and that manipulating the gut microbiota might protect against age-related cognitive dysfunction.

Because there is no direct evidence to validate that the gut microbiota mediates the effect of metformin on cognitive improvement, we cannot exclude the possibility that alteration of the microbiota caused by improvement in cognitive function or other factors contribute to the effect of metformin on cognitive improvement. Herein, we explored the underlying mechanism of the gut microbiota and specific probiotics in the effect of metformin on cognitive function in aged mice. The results showed that metformin could significantly improve the cognitive function of aged mice and alter the gut microbiota. *Akkermansia muciniphila* (*A. muciniphila*), a metformin-associated gut probiotic, improved the cognitive function of aged mice by reducing inflammation both systemically and in the hippocampus.

## Methods

### Mice and diet

Aged C57BL/6 male/female mice (postnatal age of 6–7 months) were obtained from Beijing Vital River Laboratory Animal Technology Co., Ltd. All mice were kept on a 12-h light/dark cycle with access to irradiated food and sterile water ad libitum in a temperature-controlled room with two to five mice per cage (22–25 °C). All mice were maintained in a light- and humidity-controlled climatic chamber under specific pathogen-free (SPF) conditions with fresh HEPA-filtered air. All mice had access to food and water ad libitum at a temperature of 21 °C ± 1 °C and humidity of 55% ± 10% until they are over 15 months old. After treatment, the mice underwent behavior tests or tissue collection. All animal procedures were carried out in strict accordance with the principles of laboratory animal care.

### Morris water maze (MWM) task

The MWM was used to measure hippocampal-based spatial memory and learning functions. The MWM apparatus consisted of a circular pool (1.2-m diameter) that contained water maintained at 24 to 26 °C. A clear, circular escape platform (11-cm diameter) was submerged approximately 1.5 cm below the water surface. To escape from the water, the mice had to find the hidden escape platform. Each acquisition trial was started by placing the mouse in the water facing the wall of the tank. The training protocol lasted 5 or 6 days (four trials per day). For each trial, the animal was placed in the maze near one of four possible points: north, south, southeast, or northwest. The location was determined randomly for each trial. During each trial, the animal was given 60 s to locate the submerged platform. If a mouse did not locate the platform, it was gently led to the platform. After either finding or being led to the platform, the animal was left on the platform for 20 s to allow it to become familiarized with its location with respect to visual cues. The animals were tested in squads of six to eight mice, with all treatment groups represented within each testing squad. The inter-trial interval for each mouse was approximately 20 min. The mice were subjected to probe trials 24 h after training. During the probe trials, the platform was removed and the mice were allowed to swim in the pool for 60 s. A camera mounted on the ceiling in the center of the pool was used to track the swim route of the mouse. Data were collected using a computerized animal tracking system (EthoVision XT Base; Noldus 2020 Software, Nottingham, UK), which recorded the path length, swim speed, time spent in each quadrant of the pool, and time taken to reach the platform (latency). The time spent in each quadrant was recorded. Trials were consistently performed between 1:00 pm and 5:00 pm.

### Novel object recognition (NOR) test

The NOR test takes advantage of a rodent’s spontaneous preference to explore novel objects relative to familiar objects. This is the benchmark test for recognition memory in rodents and is dependent on the integrity of the hippocampus. The test was conducted in a black soundproof chamber (35 × 40 × 40 cm). A video camera connected to a computer was used to record the exploration and testing sessions. A single trial consisted of a familiarization phase (encoding) followed by a 1-h delay interval and then a test phase (retrieval). During the familiarization phase, the mouse was allowed to explore and become familiar with two identical objects placed side-by-side in the chamber for 10 min for 3 consecutive days. A single 10-min familiarization phase was presented before the test phase. We tested the mice after a delay of 1 h. Before the test phase, one object was replaced by an object unfamiliar to the mouse (novel object). During the test phase, the mouse was allowed to explore the two objects placed side-by-side (one novel object) for 5 min. The experimenter recorded the object exploration time until the mouse touched the novel object (nose within 1 cm of the object). The novel object and the position of the novel object (left/right) were counterbalanced within each group. The experimenter was blinded to the group allocation of mice during the offline data analysis.

### Open-field test

The open-field test was performed to analyse anxiety and depression-like behaviour in mice in an open-field environment. The open field apparatus (MedAssociates, Inc., Vermont, USA) was a box (30 cm × 30 cm × 21 cm) placed in a soundproof environment with controlled lighting (approximately 200 lx). A video camera connected to a computer was used to record the exploration and testing sessions. Mice were placed along the edge of the arena and allowed to freely explore the area for 20 min. The total distance travelled, distance travelled in the central zone (10 cm × 10 cm square area), times the central zone was crossed, and time spent in the central zone were recorded using Activity Monitor software (MedAssociates, Inc., Vermont, USA).

### Antibiotic (Abx) treatment and fecal transplants

Commensal bacteria are required for normal development, especially of the immune and nervous systems; we used a model of Abx-induced dysbiosis according to a previously published protocol [28]. The Abx compounds were applied via the drinking water for 7 days and consisted of ampicillin (1 g/L; Pfizer, New York, NY, USA), vancomycin (250 mg/L; Sigma-Aldrich, St. Louis, MO, USA), neomycin (1 g/L; Sigma-Aldrich, St. Louis, MO, USA), and metronidazole (1 g/L; Sigma-Aldrich, St. Louis, MO, USA). At 72 h before gut flora reconstitution, the Abx treatment was discontinued and replaced by sterile tap water. Fresh fecal samples were collected from the same littermates of the SPF control mice and homogenized in pre-reduced phosphate-buffered saline (PBS) at 10 mL/g, vortex for 3 min, and allowed to settle for 2 min. The supernatant was filtered with a 70-μm sterile filter membrane. Finally, 200 μL of the suspension was administered by oral gavage to Abx-pretreated SPF mice. For mock treatment, mice were administrated with pre-reduced PBS.

### Bacterial culture and treatment

*A. muciniphila* (ATCC BAA835) was cultured under anaerobic conditions at 37 °C in Brain Heart Infusion media supplemented with 0.05% hog gastric mucin-type III (Sigma-Aldrich, St. Louis, MO, USA) [29]. *A. muciniphila* was freshly cultured in anaerobic conditions as described above for about 1 week and then washed, pelleted, and stored at − 80 °C with 30% glycerin. A representative glycerol stock was thawed under anaerobic conditions to determine the CFU/ml by plate counting using mucin media containing 1.5% agarose (agar noble; Difco). And samples were then resuspended at 2 × 10^9^ cfu/mL in pre-reduced PBS. Additionally, an identical quantity of *A. muciniphila* was inactivated by pasteurization for 30 min at 70 °C. Exogenous bacteria cannot be easily colonized into the mouse gut without Abx pretreatment [30]; thus, Abx-pretreated mice were gavaged every twice a day for 2 months with a 200-μl bacterial suspension, pasteurized *A. muciniphila* or sterile pre-reduced PBS with the corresponding concentration of the glycerin solution as vehicle control.

### Measurement of plasma cytokines and tissue harvesting

The mice were deeply anesthetized using tribromoethanol (Sigma-Aldrich, St. Louis, MO, USA). Blood was taken by removal eyeball and centrifuged at 1000 × g/min for 10 min at 4 °C to obtain plasma for measurement cytokines using the Bio-Plex Pro Mouse Cytokine Standard 23-Plex (Bio-Rad) according to the manufacturer’s protocol. The cytokine such as tumour necrosis factor (TNF)-α, IL-1β, and IL-6 was secondly validated with the ELISAs reagents (Absin, Shanghai, SH, China) and performed according to the manufacturer’s instructions. Cytokine concentrations are expressed in pg/mL. The mice underwent cardiac perfusion with 0.01 M PBS (Sigma-Aldrich, St. Louis, MO, USA). The brain hemispheres were placed on an ice-cold glass dissection plate and orientated in the sagittal plane. The right hemispheres were placed in 4% paraformaldehyde for cryostat sectioning and immunohistochemistry and stored at − 80 °C until required.

### Immunostaining

Brain tissue slices (10 μm) or cultured cell samples were treated with 4% PFA for 20 min and stored at room temperature. After three washes with PBS, all slides were incubated in blocking buffer (3% bovine serum albumin, 5% normal donkey serum, and 0.3% Triton X-100 in PBS) for 1 h at room temperature. Slides were immersed in primary antibody buffer (200 μL per slide) for incubation overnight at 4 °C. Primary antibodies used throughout this study include anti-Iba1 (1:500; Abcam, Cambridge, MA). The following day, coverslips and slides were washed three times with PBS and then incubated with appropriate Alexa488-secondary antibodies (1:1000; Thermo Fisher Scientific, Waltham, MA) for 1 h at room temperature. DAPI staining was used to label the nuclei for 15 min.

### Quantification of immunohistochemical staining

Each experimental group contained at least three mice. Twelve serial sections (sagittal, 10 μm) per mouse were chosen for subsequent immunostaining, according to similar anatomical locations for each mouse.

### Fecal microbiota composition (16S ribosomal RNA gene sequencing)

Fresh fecal specimens were harvested, snap-frozen, and stored at − 80 °C prior to analysis. DNA from the fecal specimens was extracted with a TIANamp Stool DNA Kit (TIANGEN, Beijing, China) according to the manufacturer’s instructions. The V3–V4 hypervariable region of the 16S ribosomal RNA gene was amplified and prepared for sequencing as outlined in the Illumina 16S Metagenomic Sequencing Library Protocol (Illumina, San Diego, CA, USA). Samples were sequenced at Teagasc Sequencing Facility on the Illumina MiSeq platform (Illumina) using a 2 × 250 bp kit. FLASH was used to assemble paired-end reads. Further processing of paired-end reads, including quality filtering based on a quality score of > 25 and removal of mismatched barcodes and sequences below the length thresholds, was completed using QIIME (version 1.9.0). Denoizing, chimera detection, and clustering into operational taxonomic unit groupings were performed using USEARCH v7 (64-bit). The operational taxonomic units were aligned using PyNAST, and taxonomy was assigned using BLAST against the SILVA SSURef database release 123. Alpha and beta diversities were generated in QIIME.

### *A. muciniphila *quantification

The relative abundance of *A. muciniphila* in feces was detected by qPCR. The primers and probes used to detect *A. muciniphila* were as described by Everarda et al. [31]: forward *A. muciniphila*, CAGCACGTGAAGGTGGGGAC; reverse *A. muciniphila*, CCTTGCGGTTGGCTTCAGAT. Detection was achieved with an ABI 7500 real-time PCR system and software (Applied Biosystems, Foster City, CA, USA). Each assay was performed at least twice in the same run. The cycle threshold of each sample was then calculated based on the standard curve created by diluting genomic DNA. The data are expressed as the fold change of *A. muciniphila* relative to the control group.

### RNA-sequencing (RNA-Seq)

The blood and hippocampus total RNAs were extracted from mice that *A.muciniphila* was not successfully colonized in the gut using the Invitrogen TRIzol following the manufacturer’s instructions. Blood and hippocampus three replicate RNA-seq libraries were prepared from the control and WAC mice groups respectively. The six libraries were performed by BGI America (Cambridge, MA) using the BGISEQ-500 sequencer. Raw sequencing reads were cleaned by removing reads containing adaptor sequences, reads containing poly-N sequences, and low-quality base ratio. Afterwards clean reads were obtained and stored in FASTQ format. Clean reads were mapped to the reference genome using HISAT2 (v2.0.4)/Bowtie2 (v2.2.5) tool. The gene expression level was calculated and normalized by RESM software. Differentially expressed genes (DGEs) analysis under the conditions of Fold Change ≥ 1.5 and FDR ≤ 0.05. The heatmap was drawn by Graphpad prism 8 according to the gene expression in different samples. To take insight to the change of phenotype, KEGG (https://www.kegg.jp/) enrichment analysis of annotated differently expressed gene was performed by Phyper (https://en.wikipedia.org/wiki/Hypergeometric_distribution) based on Hypergeometric test. The significant levels of terms and pathways were corrected by *Q* value with a rigorous threshold (*Q* value ≤ 0.05) by Bonferroni.

### Graphics

Unless otherwise specified, plots were generated using R (https://www.R-project.org/) or GraphPad Prism 8 (GraphPad Software, San Diego, CA).

## Results

### Metformin improved cognitive function and reprogramed the gut microbiota in aged mice

**Fig. 1:**
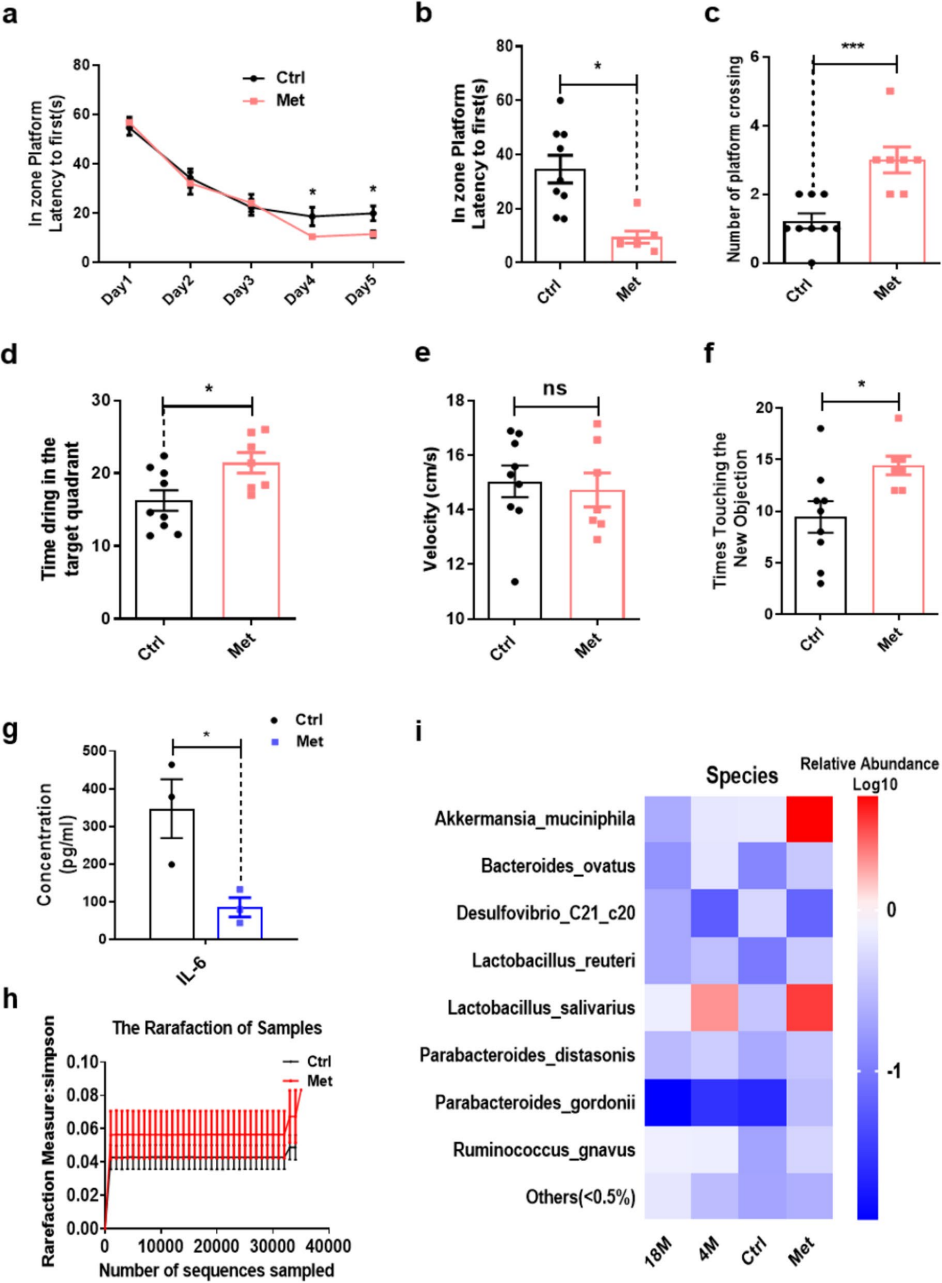
Detection of changes in cognitive function and gut microbiota in aged mice treated with metformin. **a** The escape latency was significantly decreased in the metformin-treated group during the acquisition phase. **b** The latency to reaching the former platform location was significantly decreased in the metformin-treated group. **c** The number of times the former platform location was crossed and **d** the time of staying the previous platform quadrant were significantly increased in the metformin-treated group during the probe test. **e** The mean velocity was not different between the two groups. **f** In the novel object recognition test, the number of times that a new object was touched was significantly increased in the metformin-treated group. **g** IL-6 protein was significantly decreased in the metformin-treated group. **h** The α diversity according to the Simpson index was increased after metformin treatment. **i** Heatmaps indicate the gut microbiota at the species level in metformin-treated groups is in favour of a younger phenotype (4 M) but not the older phenotype (18 M). Colours on the heatmap indicate the relative abundance of gut microbiota. Red indicates upregulated bacteria, and blue indicates downregulated bacteria. Ctrl, control; Met, metformin. The overall significance between the two groups was determined by Student’s *t*-test. *p < 0.05, **p < 0.01, ***p < 0.001, ns, not significant

To explore the effects of metformin treatment on cognitive function, the MWM and NOR test were used to examine learning and memory function. The MWM results showed that spatial learning and memory function were significantly improved in the metformin-treated group. During the 5-day learning phase, the latency to the escape platform was significantly shorter in the treatment group than that in the control group (Fig. 1a). During the probe trial, the latency to first crossing the former platform location was significantly shorter in the treatment group than that in the control group (Fig. 1b). Additionally, the number of times the mouse crossed the former location of the platform and the time in the target quadrant were greater in the treatment group than those in the control group (Fig. 1c, d). The swim velocity in the probe trial was not different between the two groups (Fig. 1e). Next, we examined working memory function using the NOR test. The results showed that the number of times mice touched the new objects was significantly increased in the treatment group (Fig. 1f). To exclude interference by depression in the evaluation of working memory, we conducted an open-field test. The results showed no difference in the number of times the centre area was crossed and in the time spent in the centre area (Fig S1a–b). These results indicated that metformin significantly improved cognitive function in aged mice.

We also detected cytokines in the plasma using an ELISA (ELISA). The results showed that the level of the inflammatory marker IL-6 was decreased in the plasma (Fig. 1g). In addition, to investigate the improvement of cognitive function and the decrease in systemic proinflammatory cytokines, we also detected the microbial community structure after metformin treatment for 1 month. In a metagenomic analysis of stool samples from mice treated with metformin for 1 month, the α diversity according to the Simpson index indicated that metformin-treated mice exhibited different gut microbiota structures (Fig. 1h). At the species level, dramatic increases were observed in the relative abundances of *A. muciniphila, Lactobacillus salivarius, Lactobacillus reuteri,* and *Parabacteroides distasonis* in aged mice treated with metformin (Fig. 1i). These members of the gut microbiota have been reported to be associated with other disorders. To further screen organisms in the gut microbiota associated with ageing, we detected the microbial community structure in 4-month-old and 18-month-old mice and observed changes in the relative abundance of *A. muciniphila*, *Lactobacillus salivarius*, *Bifidobacterium pseudolongum*, *Bacteroides ovatus*, *Lactobacillus reuteri*, and *Parabacteroides distasonis,* which were sharply decreased in aged mice (Fig. 1i). These results reveal that metformin might alter the gut microbiota structure in favour of a younger phenotype.

### The gut microbiota is necessary and sufficient for the effects on cognitive function induced by metformin

**Fig. 2:**
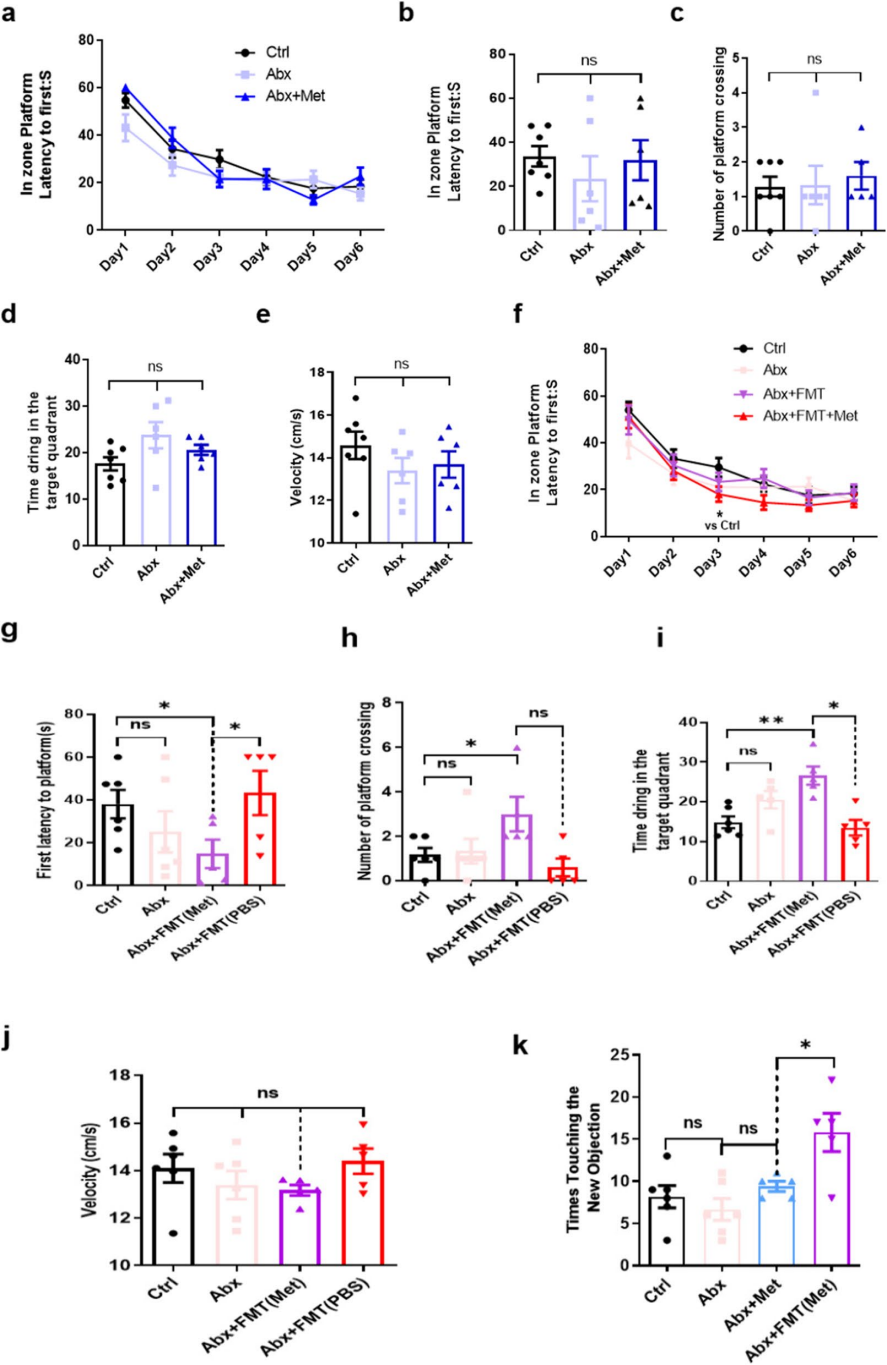
The gut microbiota is necessary and sufficient for the effects of metformin on cognitive function. **a** The escape latency in the Abx and metformin-treated (Abx + Met) group was not different from that of the Abx-treated and control groups during the acquisition phase. **b** Latency to first reaching the platform. **c** Number of times mice crossed the platform. **d** Time that mice stay in target quadrant where the platform was located. **e** The mean velocity was not different among the Abx + Met, Abx, and control groups. **f** The FMT + Met group had a shorter latency in reaching the escape platform than the control, Abx-treated, and FMT-treated groups during the acquisition phase. **g** The latency to first crossing the previous platform location was significantly shorter in the FMT + Met group than that in the control, Abx, and FMT groups. **h** The number of times the previous platform location was crossed and **i** the time in the target quadrant were significantly increased in the FMT + Met group during the probe test. **j** The mean velocity during the probe test was not different among the four groups. **k** The number of new objects touched times was significantly increased in mice treated with metformin and decreased in the Abx + Met group. However, this behaviour was restored in Abx-treated specific pathogen-free mice who underwent standardization of the gut microbiota with that from mice in the same litter. Ctrl, control; Met, metformin. The overall significance among three or four groups was determined by one-way ANOVA. *p < 0.05, **p < 0.01, ***p < 0.001, ns, not significant

To determine whether the gut microbiota is necessary for the improvement of cognitive function induced by metformin, we measured the cognitive function of aged mice treated with metformin. SPF mice treated with metformin for 1 month exhibited cognitive function improvement and alteration of the microbiota (Fig. 1a, j). Meanwhile, we noticed that Abx treatment of mice for 3 or 6 days eliminated a large proportion of the gut microbiota, and the α diversity index, ace index, observed_species, and Chao index were significantly decreased (Fig S2a–c). Mice pre-treated with Abx for 3 days were considered germ-free, and these mice were then treated with metformin. The MWM and NOR results showed that Abx treatment of SPF mice prevented the improvements of spatial memory and working memory function induced by metformin (Fig. 2a, k). The metformin-induced decrease in latency to the escape platform during the 5-day learning phase compared with that in the control group was abolished (Fig. 2a). Additionally, the number of times the former platform location was crossed and the time in the target quadrant were also similar to those of the control group during the probe trial (Fig. 2b–d). The swim velocity in the probe trial was not different among the three groups (Fig. 2e). The results indicated that the gut microbiota is necessary for metformin-mediated improvement of spatial memory function. Postnatal standardization of the gut microbiota via faecal microbiota transplantation (FMT) of gut microbiota from mice in the same litter restored cognitive function in Abx-treated SPF mice to levels seen in naïve SPF metformin-treated mice. Mice in the FMT + Met treatment group exhibited a shorter latency to the escape platform than those in the control, Abx-treated, and FMT-treated groups during the 5-day learning phase (Fig. 2f). Additionally, the latency to crossing the former platform location was significantly shorter in the FMT + Met treatment group than that in the control, Abx-treated, and FMT-treated groups (Fig. 2g). Furthermore, the number of times the former platform location was crossed and the time in the target quadrant were significantly increased in the FMT + Met group during the probe trial (Fig. 2h–i). The swim velocity in the probe trial was not different among the four groups (Fig. 2j), suggesting that the gut microbiota is required for the effects of metformin on cognitive function improvement in aged mice. The NOR experiment results also showed that the number of times mice touched new objects was significantly increased among mice treated with metformin. The effect of metformin on improving working memory function was abrogated in Abx treatment of SPF mice, and this effect was restored after standardization of the gut microbiota in Abx-treated SPF mice to that from mice in the same litter (Fig. 2k). These results indicated that the gut microbiota is necessary and sufficient for the effects of metformin.

### *A. muciniphila*, which is enriched in the gut microbiota by metformin, improves cognitive function in aged mice

**Fig. 3:**
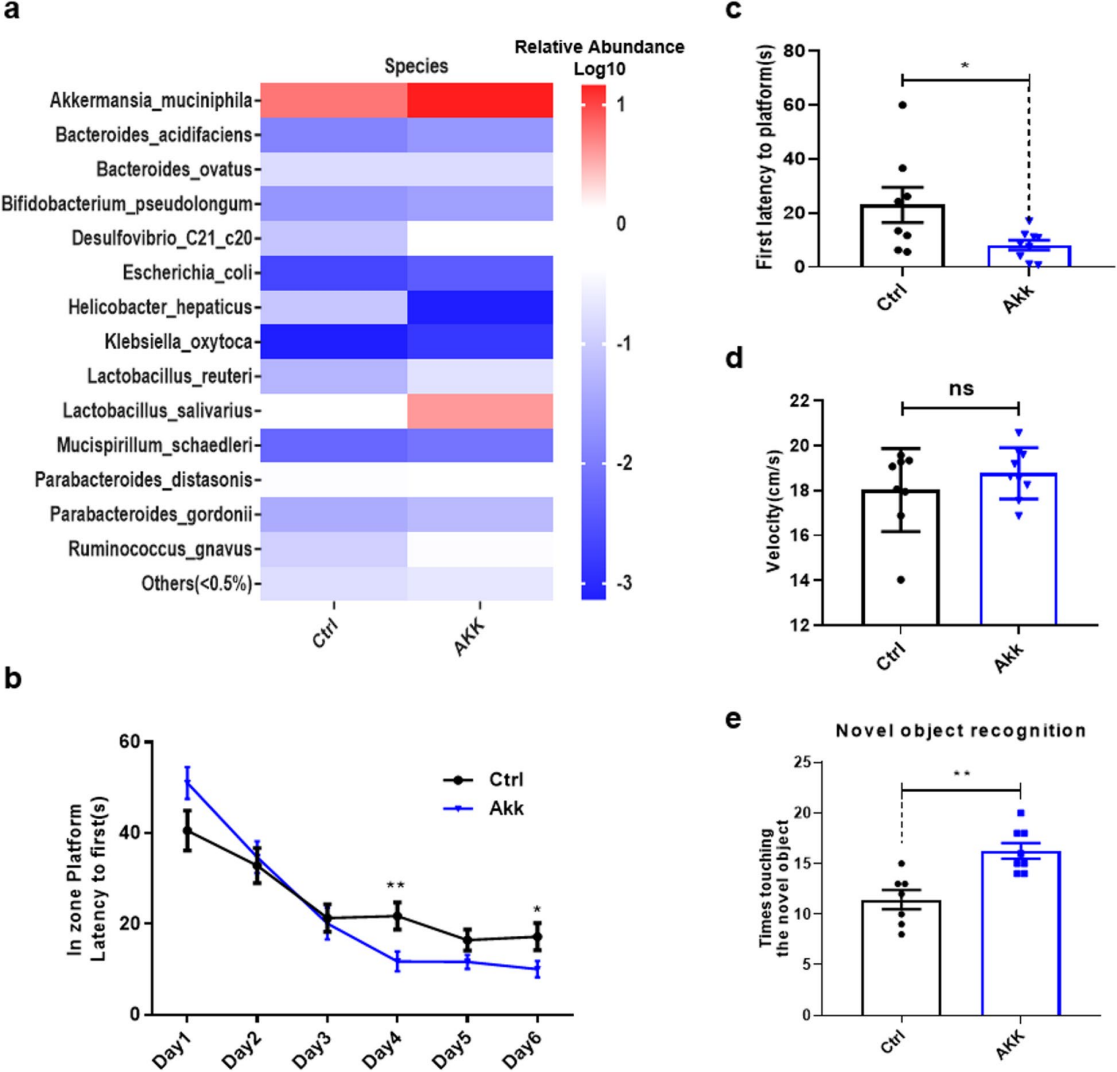
*A. muciniphila* improved cognitive function in aged mice. **a** Heatmap of significant changes in the gut microbiota at the species level after *A. muciniphila* treatment. Colours on the heatmap indicate the relative abundance of gut microbiota. Red indicates upregulated bacteria, and blue indicates downregulated bacteria. **b** The escape latency in the *A. muciniphila*-treated mouse group was significantly shorter than that in the control group during the acquisition phase. **c** The latency to first reaching the platform in the *A. muciniphila*-treated group was significantly shorter than that in the control group during the probe trial. **d** The mean swimming velocity was not different between the *A. muciniphila-*treated and control groups during the probe trial. **e** Times of the new object touched were significantly increased in the *A. muciniphila* group compared with that in the control group. Ctrl, control; AKK, *A. muciniphila*. The overall significance between the two groups was determined by Student’s *t*-test. *p < 0.05, **p < 0.01, ***p < 0.001; ns, not significant

We aimed to determine whether specific bacterial taxa mediate cognitive function improvement in response to metformin. *A. muciniphila* was the first taxa found to be enriched by metformin, and its level decreases during ageing (Fig. 1). *A. muciniphila* is a commensal organism that resides in the mucus layer of the gut and plays a key role in degrading mucin [29, 32, 33]. However, the influence of *A. muciniphila* on the host physiological functions is controversial [34]. Some researchers have reported that an abundance of *A. muciniphila* was inversely correlated with body weight, fat mass, inflammation, insulin resistance, and glucose intolerance [31, 35–37]. However, proof-of-concept studies have been conducted, and in some studies, over-representation of *A. muciniphila* in the faces was not associated with a beneficial effect. Other studies have reported an increased abundance of *A. muciniphila* with ingestion of a high-fat, high-sucrose diet and in patients with diabetes [38]. Correspondingly, an increased abundance of *A. muciniphila* has also been found in a series of studies investigating Parkinson’s disease and multiple sclerosis [39–42]. To determine whether *A. muciniphila* mediates cognitive function improvement in response to metformin, mice were gavaged with 10^9^ cfu *A. muciniphila* every 2 days for 30 days. After oral gavage, mice treated with *A. muciniphila* harboured a higher relative abundance of *A. muciniphila* (Fig. 3a). We next examined cognitive function in mice treated with *A. muciniphila* using the MWM and NOR test. The results showed that cognitive function was significantly improved in the *A. muciniphila-*treated group. The MWM results showed that the latency to reaching the escape platform was significantly shorter in the treatment group than that in the control group during the 5-day learning phase (Fig. 3b). The latency to first crossing the previous platform location (Fig. 3c) was significantly shorter in the treatment group than that in the control group during the probe trial, and the swim velocity in the probe trial was not different between the two groups (Fig. 3d). The NOR test results showed that the number of times new objects were touched was significantly increased in the treatment group (Fig. 3e). Additionally, no difference was observed in times of the centre area crossed, the time spent in the centre area, and the ambulatory distance in the open field test (Fig S3a–c). These results suggest that *A. muciniphila*, which is enriched by metformin, improves cognitive function.

### *A. muciniphila* downregulated pro-inflammatory pathways in the peripheral blood and decreased pro-inflammatory cytokine levels in the plasma

**Fig. 4:**
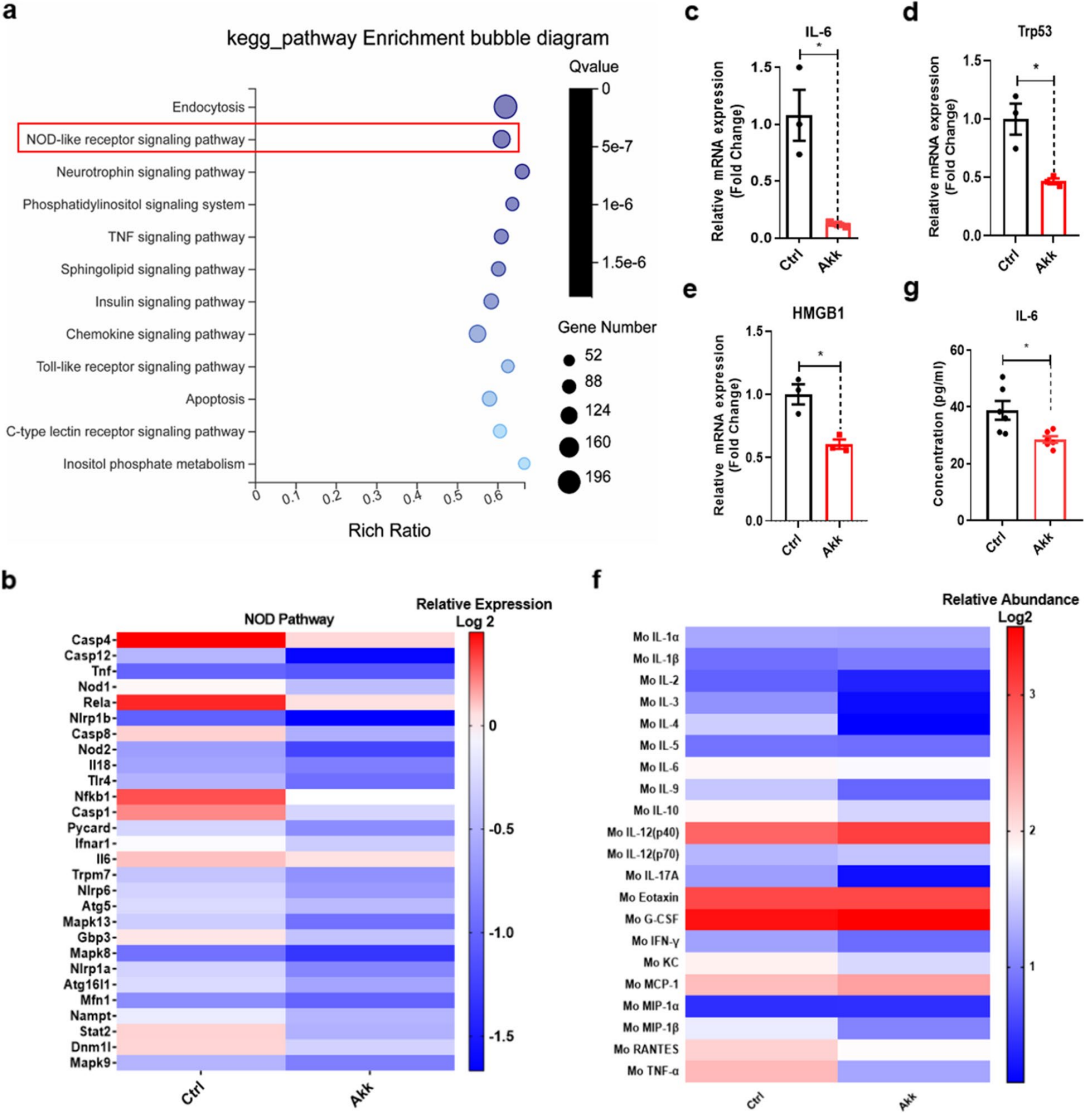
*A. muciniphila* downregulated inflammatory pathways and proinflammatory cytokine expression in the blood. **a** Pathway enrichment analysis using RNA sequencing indicated genes that were downregulated in the blood of aged mice treated with *A. muciniphila* compared with those in the blood of control mice. **b** Expression heatmap of the top DGEs in the NOD-like receptor signalling pathway from the blood of aged mice treated with *A. muciniphila* compared with those in the control group. **c**–**e** Quantitative reverse-transcription polymerase chain reaction results validated that the relative mRNA expression levels of IL-6, Trp53, and Hmg proteins were significantly decreased in the *A. muciniphila-*treated group. **f** Effect of *A. muciniphila* on plasma cytokine proteins of aged mice as detected by a cytokine antibody array (*n* = 5–6). Colours on the heatmap indicate relative cytokine levels. Red indicates upregulated cytokines, and blue indicates downregulated cytokines. **g** An ELISA verified that the key proinflammatory cytokine IL-6 was significantly decreased after *A. muciniphila* treatment. Ctrl, control; AKK, *A. muciniphila*. The overall significance between the two groups was determined by Student’s *t*-test. *p < 0.05, **p < 0.01, ***p < 0.001; ns, not significant

We showed that *A. muciniphila* treatment significantly improved cognitive function in aged mice (Fig. 3a, e), but the underlying mechanism has not been elucidated. Therefore, we explored this mechanism. Peripheral blood from mice treated with *A. muciniphila* was subjected to transcriptome analysis to determine the potential molecular mechanism by which *A. muciniphila* ameliorates cognitive impairment during ageing. The analysis identified 8003 DGEs, including 7966 downregulated and 37 upregulated genes (Fig S4a). Analysis of differential gene expression in the blood clearly demonstrated that, compared with those in the control group, the NOD-like receptor, tumour necrosis factor (TNF), T cell receptor, chemokine, insulin, mammalian target of rapamycin (mTOR), and C-type lectin receptor signalling pathways were significantly downregulated in the *A. muciniphila*-treated group (Fig. 4a). Additionally, the mTOR and insulin signalling pathways are considered classical anti-ageing signalling pathways [43]. Of note, genes related to the NOD-like receptor were significantly downregulated (Fig. 4b). Meanwhile, IL-6, Trp53, and the high mobility group (Hmg) protein family, which is involved in ageing and neurodegeneration via pro-inflammatory activity [44, 45], were downregulated in the *A. muciniphila*-treated group (Fig. 4c–e). To confirm the results, plasma cytokine and chemokine levels were compared between the *A. muciniphila*-treated and control groups using the Bio Plex Pro Mouse Cytokine Grp Panel 23 plex (M60009RDPD). The results showed that proinflammatory cytokines, including IL-6, TNF-α, and IL-1β, were downregulated in the *A. muciniphila*-treated group (Fig. 4f). Several meta-analyses of prospective studies have demonstrated that the peripheral inflammatory factor IL-6 is associated with cognitive decline in adults without dementia [46]. Therefore, an ELISA was performed to further validate the expression level of IL-6. The results confirmed that the plasma IL-6 protein level was significantly reduced (Fig. 4g), and we found that IL-1β and TNF-α were also downregulated (Fig S4b–c). These results demonstrated that *A. muciniphila* suppresses proinflammatory pathways in the blood, especially IL-6 expression levels.

### *A. muciniphila* suppressed inflammatory pathways and stimulated the neuroactive pathway in hippocampal tissue of aged mice

**Fig. 5:**
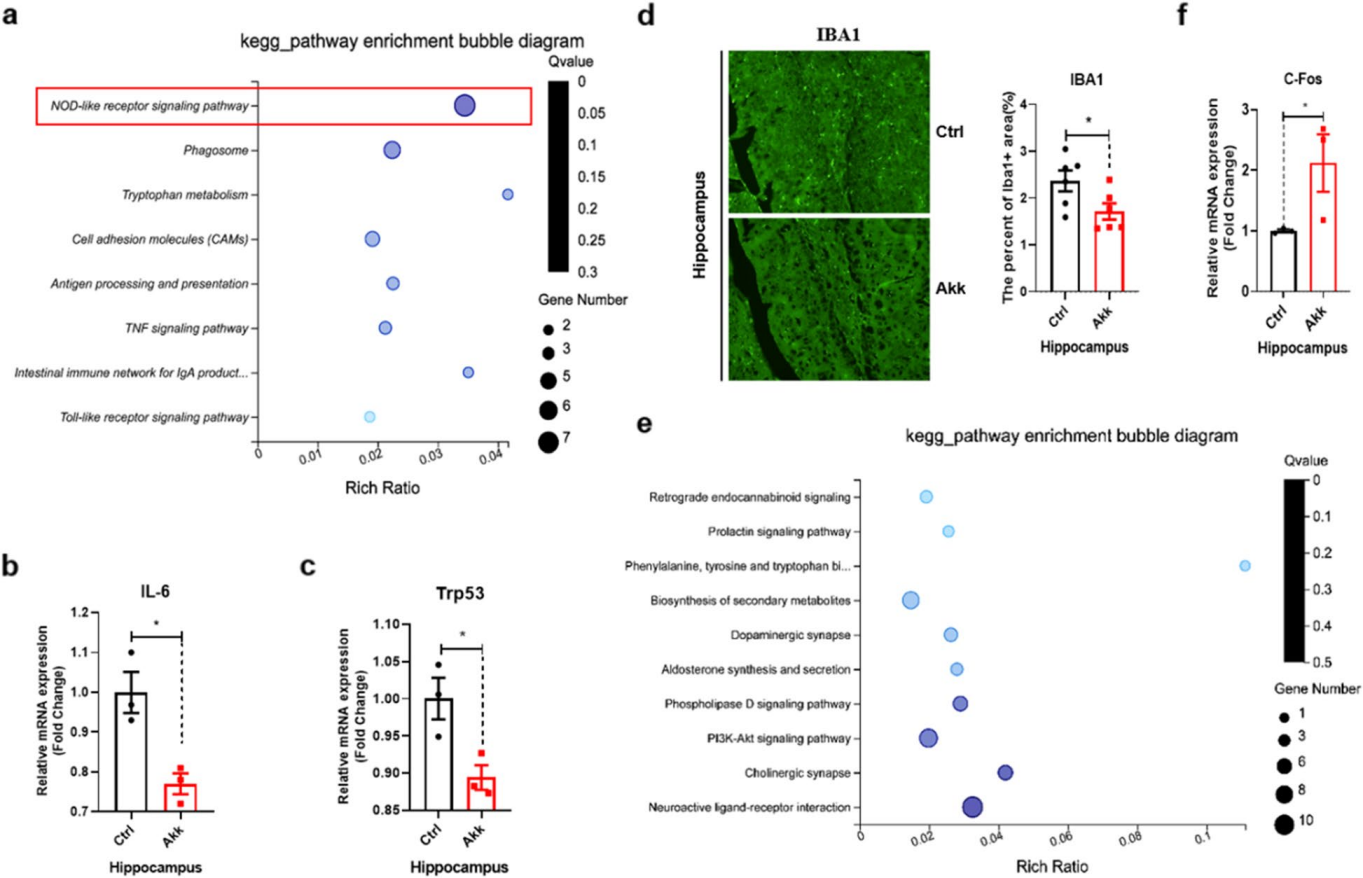
*A. muciniphila* downregulated inflammatory and upregulated neuroactive pathways in hippocampal tissue. **a** Pathway enrichment analysis using RNA sequencing showed downregulated expression of genes in the hippocampus in *A. muciniphila-*treated mice. **b**, **c** Quantitative reverse-transcription polymerase chain reaction PCR results confirmed that the relative mRNA expression levels of IL-6 and Trp53 in hippocampal tissue were significantly decreased in the *A. muciniphila-*treated group. **d** *A. muciniphila* significantly decreased the amount of microglia compared with that in the control group. **e** Pathway enrichment analysis using RNA-seq showed upregulation of gene expression in the hippocampus of aged mice treated with *A. muciniphila* compared with that in the control group. **f** *A. muciniphila* upregulated mRNA expression of the neuronal transcription factor c-Fos. Ctrl, control; AKK, *A. muciniphila*. The overall significance between the two groups was determined by Student’s *t*-test. *p < 0.05, **p < 0.01, ***p < 0.001; ns, not significant

The CNS and peripheral immune system, two major physiological systems, communicate with each other constantly and extensively through multiple pathways. Additionally, they are involved in the modulation of behaviour and many other critical neurological functions throughout life [16, 47]. Next, we explored transcriptome changes in hippocampal tissue in the *A. muciniphila* group. In total, 337 DGEs were identified, including 155 downregulated and 182 upregulated genes (Fig S5a). Differential expression analysis of downregulated genes in the hippocampal tissue clearly demonstrated that compared with that in the control group, *A. muciniphila* exhibited decreased expression of inflammatory pathways in hippocampal tissue, including the NOD-like receptor, TNF, chemokine, and mTOR signalling pathways, similar to those in the peripheral blood (Fig. 5a). The IL-6 and Trp53 mRNA expression levels were also downregulated by *A. muciniphila* treatment (Fig. 5b, c). Microglia, as immune cells in the brain, are constantly activated during ageing [48]. In this study, immunofluorescence staining demonstrated that microglia (Iba1^+^ cells) were hyperactive during ageing (Fig S5b) and that *A. muciniphila* treatment significantly decreased the number of microglia in aged mice (Fig. 5d). Meanwhile, differential expression analysis of upregulated genes in hippocampal tissue demonstrated that compared with that in the control group, *A. muciniphila* increased the expression of genes involved in the neuroactive ligand-receptor interaction, cholinergic synapse, dopaminergic synapse, and prolactin signalling pathways (Fig. 5e). The relative mRNA expression level of the neuronal transcription factor c-Fos was significantly upregulated compared with that in the control group (Fig. 5f). These results demonstrated that *A. muciniphila* treatment could significantly downregulate pro-inflammatory pathways and promote neuroactivity in hippocampal tissue.

### A. muciniphila improved the cognitive function of aged mice by decreasing the level of the systemic inflammation biomarker IL-6

**Fig. 6:**
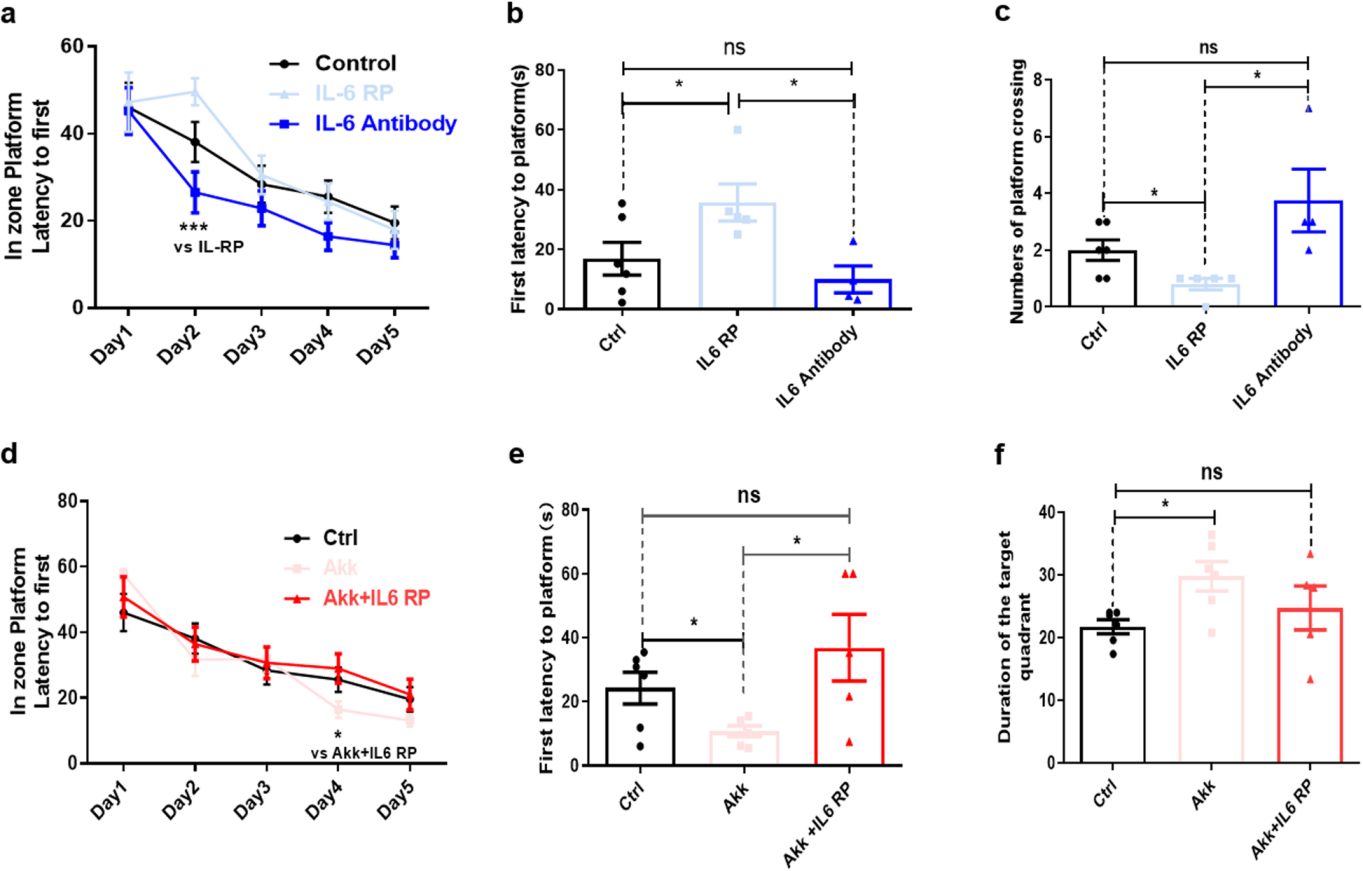
IL-6 plays a key role in *A. muciniphila-*mediated improvement of cognitive function. **a** The escape latency during the acquisition phase was significantly shorter in IL-6 antibody-treated mice than that in the control group and the IL-6 recombinant protein (RP)-treated group. **b** The latency to first crossing the former platform location was slightly decreased in the IL-6 antibody-treated group but not in the IL-6 RP-treated group during the probe test. **c** The number of times that the platform location was crossed was significantly decreased in the IL-6 RP-treated group and increased in the IL-6 antibody-treated group compared with that in the control group. **d** The escape latency was significantly shorter in the *A. muciniphila-*treated group than that in the control group, but this difference was eliminated after IL-6 RP treatment. **e** The time spent in the target quadrant during the probe trial was significantly increased in the *A. muciniphila-*treated group, but the difference was eliminated after IL-6 RP treatment. Ctrl, control; AKK, *A. muciniphila*. The overall significance among three or four groups was determined by one-way-ANOVA. *p < 0.05, **p < 0.01, ***p < 0.001; ns, not significant

Systemic inflammation increases the risks of cognitive impairment, neurological disorders, and neurodegeneration [16, 46]. Several meta-analyses of prospective studies demonstrated that the peripheral inflammation factor IL-6 is associated with cognitive decline in adults without dementia [46]. In this study, we revealed that peripheral IL-6 protein levels were increased during ageing (Fig S6a), and *A. muciniphila* treatment reversed this upregulation at both the mRNA and protein levels in aged mice (Fig. 4c, g). Thus, we hypothesized that the anti-inflammatory effects of *A. muciniphila* involved downregulation of IL-6, thereby impeding further cognitive impairment in aged mice. First, we validated the effects of elevated peripheral IL-6 levels on cognitive function in aged mice. Aged mice were treated with an IL-6 recombinant protein (IL-6 RP) and an IL-6 monoclonal antibody (IL-6 antibody) via tail vein injection, and IgG was used as a control. The MWM test was used to examine spatial learning and memory function. In the 5-day learning phase, we observed a significantly decreased platform escape latency in IL-6 antibody-treated mice but not in mice treated with IL-6 RP (Fig. 6a). During the probe trial, a significantly increased latency to first crossing the previous platform location was observed in the IL-6 RP-treated group but not in the IL-6 antibody-treated group (Fig. 6b). Additionally, the number of times the platform location was crossed was significantly decreased in the IL-6 RP-treated group and increased in the IL-6 antibody-treated group compared with that in the control group (Fig. 6c). We also noticed that the IL-6 antibody-treated group, but not the IL-6 RP-treated group, spent significantly more time in the target quadrant than the control group (Fig S6b). There was no difference in the swimming speed on day 6 of testing among the three groups (Fig S6c). These results indicate that IL-6 plays a vital role in cognitive function in aged mice. To determine whether peripheral IL-6 participates in the *A. muciniphila*-mediated effects on cognitive function, mice were treated with IL-6 RP after *A. muciniphila* pre-treatment, and the MWM test was used to assess spatial learning and memory function. In the 5-day learning phase, we found a significantly decreased latency to the escape platform in the *A. muciniphila-*treated group, whereas this difference was eliminated by IL-6 RP treatment (Fig. 6d). During the probe trial, the latency to first crossing the previous platform location (Fig. 6e) was significantly decreased in the *A. muciniphila-*treated group and increased in the IL-6 RP-treated group compared with that in the control group. The *A. muciniphila-*treated group spent significantly more time in the target quadrant during the probe trial than the control group, but this difference was eliminated by IL-6 RP treatment (Fig. 6f). Other indicators of cognitive function in the MWM test such as the number of times the previous location of the platform was crossed were slightly improved by *A. muciniphila* treatment, whereas no difference was noted in the IL-6 RP-treated group compared with the control group (Fig S6d). There was no difference in swimming speed on day 6 of testing among the three groups (Fig S6e). These results indicated that the IL-6 antibody promoted cognitive function protection, and the IL-6 RP abolished the effect of *A. muciniphila* on protection of cognitive function in aged mice.

## Discussion

Ageing is a natural process of organisms involving the decay of various physiological functions throughout life [14, 43]. Longitudinal studies indicated that most individuals develop a chronic low-grade pro-inflammatory state with ageing, which is termed inflammageing [22, 49], and such a pro-inflammatory state increases the vulnerability to various intrinsic and extrinsic disruptive effects on multi-morbidity, physical disability, frailty, and death. Considering the trillions of microbes residing in and on the human body, a growing body of evidence supports their pivotal roles in health, anti-inflammatory processes, and metabolism across life [47, 50, 51]. Age-related changes in the microbiota composition have been strongly linked to increased intestinal permeability and age-associated inflammation in the host, eventually leading to death [52–56].

Metformin plays a key role in metabolism, cancer, ageing, and neurodegenerative disease [2, 57–59]. Many studies have reported that metformin affects the host metabolism through the gut microbiota [8, 10, 13]. The microbiota plays a key role in host digestion, metabolism, and behaviour, but whether microbial responses to metformin also impact cognitive function in aged mice is poorly understood. The results from our study demonstrated that metformin shaped the gut microbiota and improved cognitive function in aged mice, and these protective effects were abrogated after Abx treatment. Postnatal FMT from mice in the same litter restored cognitive function in Abx-pretreated SPF mice to levels seen in native SPF mice treated with metformin, suggesting that the link between metformin treatment and cognitive function improvement in mice could be mediated by the microbiota. Future investigations are warranted to determine whether cognitive function in germ-free mice is associated with microbial transplantation.

We observed that metformin reduced the gut bacterial α diversity in aged mice while increasing the relative abundance of *A. muciniphila*, *Lactobacillus reuteri*, *Lactobacillus salivarius*, and *Parabacteroides distasonis*, which all decrease with age. Metformin can dramatically increase the abundance of *A. muciniphila* (up to 20% of the total microbiota) in patients with diabetes, as has been shown in many studies, and diet-induced increases in *A. muciniphila* are observed during fasting in humans [37, 60, 61]. *A. muciniphila* and *Parabacteroides* are also associated with increased ketosis [27] and metabolic improvement in humans [62]. Interestingly, the abundance of *A. muciniphila* is increased in super-centenarians [63]. Moreover, *A. muciniphila* plays a role in immune modulation [64], has been shown to protect against inflammation and promote gut health in diet-induced obesity [31], and restores intestinal permeability and subsequent immunomodulation in aged mice [65]. One study reported changes in the gut microbiota in response to a high-fat diet in C57Bl/6 J mice, and the *A. muciniphila* levels were correlated with cognitive function and reduced serum levels of inflammatory markers. One study predicted that learning performance was strongly correlated with the abundance of *A. muciniphila*. Studies have suggested that a link exists between *A. muciniphila* and cognitive performance, and the underlying mechanism needs to be clarified. The present study showed that the abundance of *A. muciniphila* was strongly decreased in 18- vs. 4-month-old mice, and this result is consistent with that of a previously reported study [24]. We also found that *A. muciniphila* could significantly enhance cognitive performance and decrease inflammation levels in aged mice. However, future studies are warranted to investigate the beneficial effects of metformin-induced enrichment of other gut microbes on cognitive performance and healthy ageing.

The CNS and peripheral immune system communicate with each other constantly and extensively through multiple pathways, and they are involved in the modulation of behaviour and other critical neurological functions throughout life [25, 66, 67]. Peripheral immunosenescence and inflammageing can promote neuroinflammation by promoting an active pro-inflammatory state in glial cells, leading to a loss of neuroprotective function, neuronal dysfunction, and accumulation of brain tissue damage [68, 69]. Our results revealed that *A. muciniphila* downregulated pro-inflammatory pathways in both the CNS and blood. Interestingly, we found that *A. muciniphila* significantly suppressed the systemic inflammatory biomarker IL-6. Several meta-analyses of prospective studies have demonstrated that peripheral IL-6 expression is associated with cognitive decline in adults without dementia, which has been identified as a blood marker of cognitive impairment and the severity of cognitive dysfunction [47, 69, 70]. Peripheral IL-6 overproduction results in an increase in the IL-6 concentration in the brain. Systemically produced IL-6 may permeate the blood–brain barrier and, after reaching the brain, might be detrimental for neurogenesis [71, 72]. IL-6 overproduction may also impair neurotransmission in brain structures which modulate cognitive functions, e.g. the hippocampus and prefrontal cortex [73, 74]. IL-6 also induced alterations of monoamine (mainly 5-HT) concentrations and glutamatergic or GABA-ergic neurotransmission release of synaptic activity in the hippocampus and frontal cortex, which may cause the bad effects on cognitive function. The blood concentration of IL-6 is also related to hippocampal grey matter volume [75]. However, blockade of IL-6 in a mouse model with loss of neurogenesis largely restored hippocampal neurogenesis [76], which indicates that peripheral IL-6 could be a useful biomarker for impaired cognitive function. Additionally, tailored or preventive strategies directed toward peripheral IL-6 may be an effective method to protect cognitive function. Here, we reported that *A. muciniphila* administration may be an effective method to improve cognitive function in older mice by reducing peripheral Il-6 production. Therefore, we treated mice with an IL-6 RP and an IL-6 antibody. The MWM test results illustrated that elevated peripheral IL-6 expression impaired the spatial learning and memory abilities of aged mice, and decreased peripheral IL-6 expression ameliorated this effect. Surprisingly, our results confirmed that IL-6 plays a key role in the *A. muciniphila*-mediated improvement of cognitive function. Naïve CD4^+^ T cells differentiate into Th17 cells under the influence of the environmental cytokines IL-6 and transforming growth factor-β [77]. With ageing, Th17 cells produce high levels of pro-inflammatory cytokines and impair cognitive function [78]. The initiation of Th17 cell differentiation requires IL-6, SMAD2, and tripartite motif-containing proteins. We found that the relative mRNA expression of Th17 cell differentiation pathway genes was significantly downregulated by *A. muciniphila* treatment [79, 80]. We hypothesized that *A. muciniphila* improves cognitive function in aged mice by inhibiting Th17 cell activity in the circulatory system and hyperactive microglia in the brain, thereby reducing the secretion of pro-inflammatory cytokines and improving cognitive function. Future research is needed to determine whether peripheral pro-inflammatory cells infiltrate the brain of aged mice and impair cognitive function. Here we reported that *A. muciniphila* administration may be an effective way to improve cognitive function in older mice by reducing peripheral IL-6 production.

## Conclusion

Taken together, we explored the underlying mechanism of the gut microbiota and specific probiotics in the effect of metformin on cognitive function in aged mice. Our results reveal that metformin could significantly improve the cognitive function of aged mice and alter the gut microbiota. *A. muciniphila*, which is mediated in the gut microbiota by metformin, modulates inflammation-related pathways in the host and improves cognitive function in aged mice by reducing the pro-inflammatory cytokine IL-6 both systemically and in the hippocampus. There is direct evidence to validate that the gut microbiota mediates the effect of metformin on cognitive improvement.

## Supplementary Information

**Additional file 1.**

## Data Availability

The original 16S rRNA sequence data are available at the NCBI by accession number PRJNA655975. The RNA-seq data files that were generated in this study are available from the NCBI Gene Expression Omnibus (GEO) under the accession number GEO: GSE162123.

## Abbreviations

IL-6: Interleukin-6
CNS: Central nervous system
A. muciniphila: Akkermansia muciniphila
SPF: Specific pathogen-free
MWM: Morris water maze
NOR: Novel object recognition
Abx: Antibiotic
TNF-α: Tumour necrosis factor α
PBS: Phosphate-buffered saline
RNA-Seq: RNA-sequencing
DGEs: Differentially expressed genes
ELISA: Enzyme-linked immunosorbent assay
FMT: Faecal microbiota transplantation
mTOR: Mammalian target of rapamycin
Hmg: High mobility group
