# Supplementary material for: Akkermansia muciniphila, which is enriched in the gut microbiota by metformin, improves cognitive function in aged mice by reducing the proinflammatory cytokine interleukin-6
Source: Microbiome. 2023 May 30;11:120. doi: 10.1186/s40168-023-01567-1 (PMC10228018; doi:10.1186/s40168-023-01567-1)
Supplement: Supplementary file 2 — Additional file 1. [file 40168_2023_1567_MOESM1_ESM.docx]

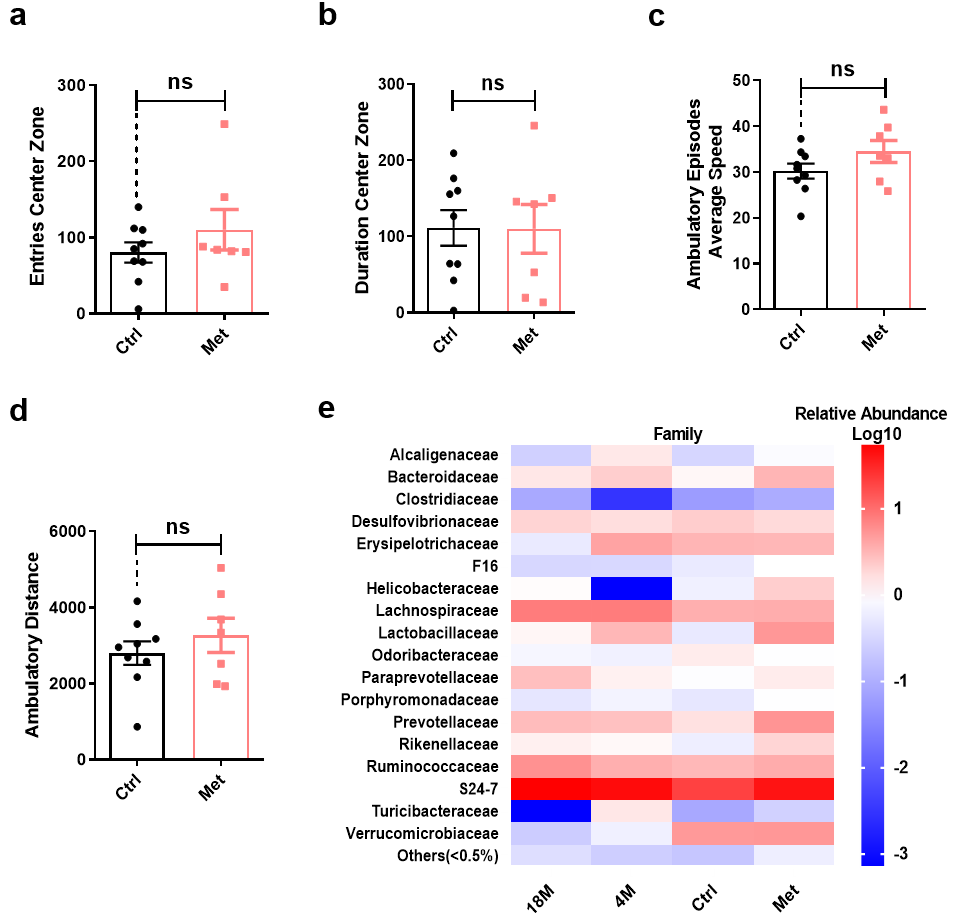


**Fig S1. Anxiety and depression indicated by the open field test and changes in gut microbiota after metformin treatment.** (a) The number of entries into the centre zone, (b) duration in the centre zone, (c) average speed of ambulatory episodes, and (d) ambulatory distance were not different in the control and metformin-treated groups. (e) Heatmaps indicate changes in the gut microbiota structure of metformin treatment at the family is in favour of a younger phenotype (4M) but not the older phenotype (18M). Colours on the heatmap indicate the relative abundance of gut microbiota. Red indicates upregulated bacteria, and blue indicates downregulated bacteria. Ctrl: Control; Met: Metformin. The overall significance between two groups was determined by Student’s *t*-test. * *p* < 0.05, ** *p* < 0.01, *** *p* < 0.001, ns, not significant.


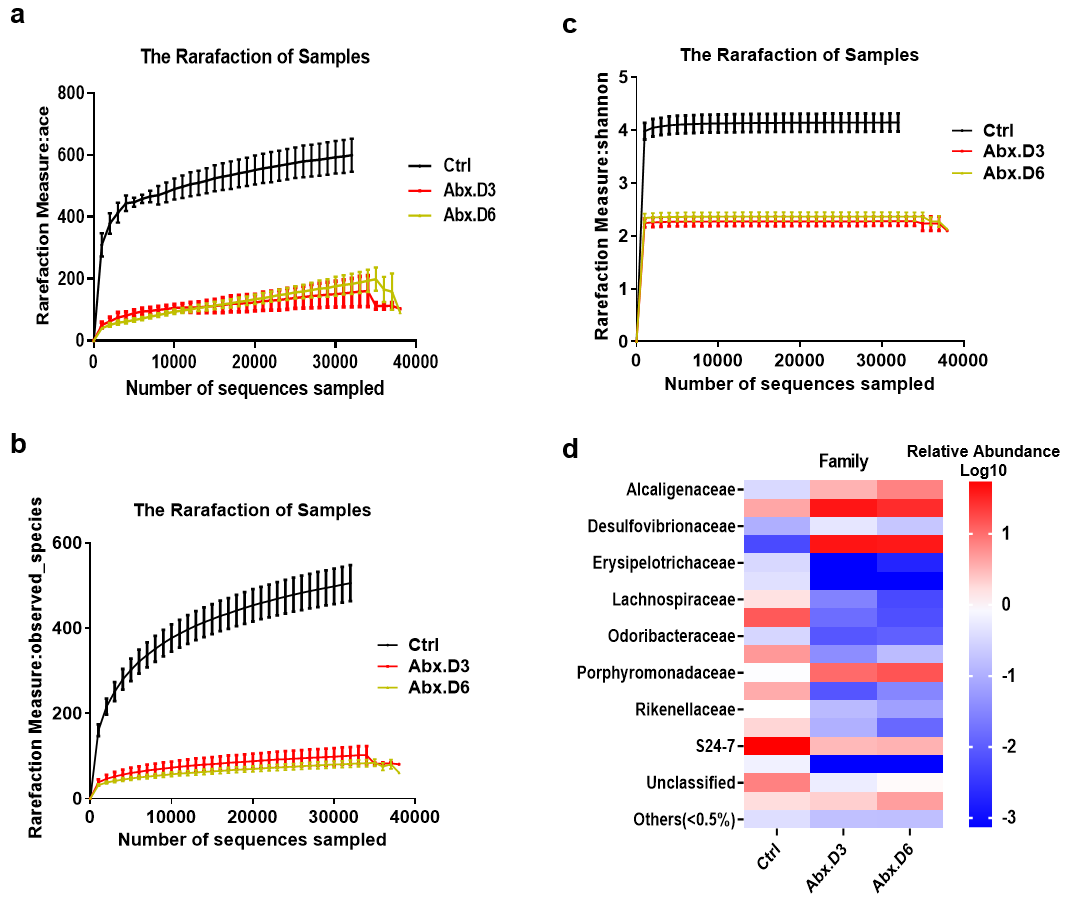


Fig S2. The gut microbiota was eliminated in aged mice treated with Abx for 3 or 6 days. (a, b, c) α Diversity indicators, including the ace index, observed_species, and shannon index, were significantly decreased after Abx treatment for 3 or 6 days. (d) The heatmap shows significant gut microbiota structure changes at the family level after Abx treatment for 3 or 6 days. Colours on the heatmap indicate the relative abundance of gut microbiota. Red indicates upregulated bacteria, and blue indicates downregulated bacteria.


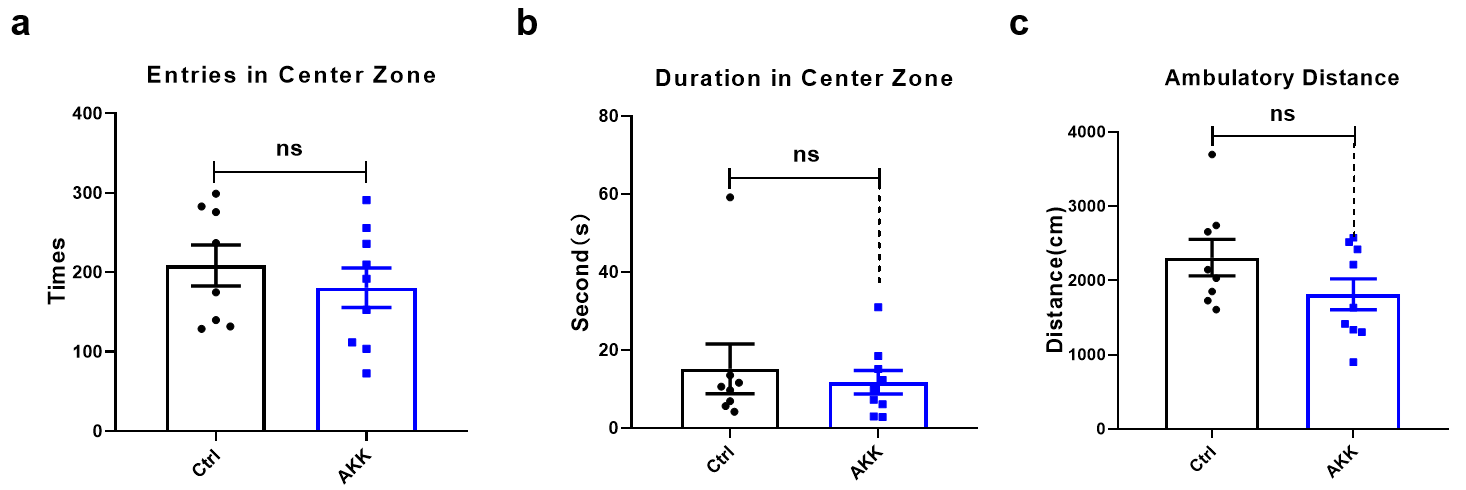


**Fig S3. Open field test analysis of anxiety and depression after *A. muciniphila* treatment.** (a) Entries into the centre zone. (b) Duration in the centre zone. (c) The ambulatory distance was not different between the control and *A. muciniphila*-treated groups. Ctrl: Control; AKK: *A. muciniphila*. The overall significance between two groups was determined by Student’s *t*-test. * *p* < 0.05, ** *p* < 0.01, *** *p* < 0.001, ns, not significant.


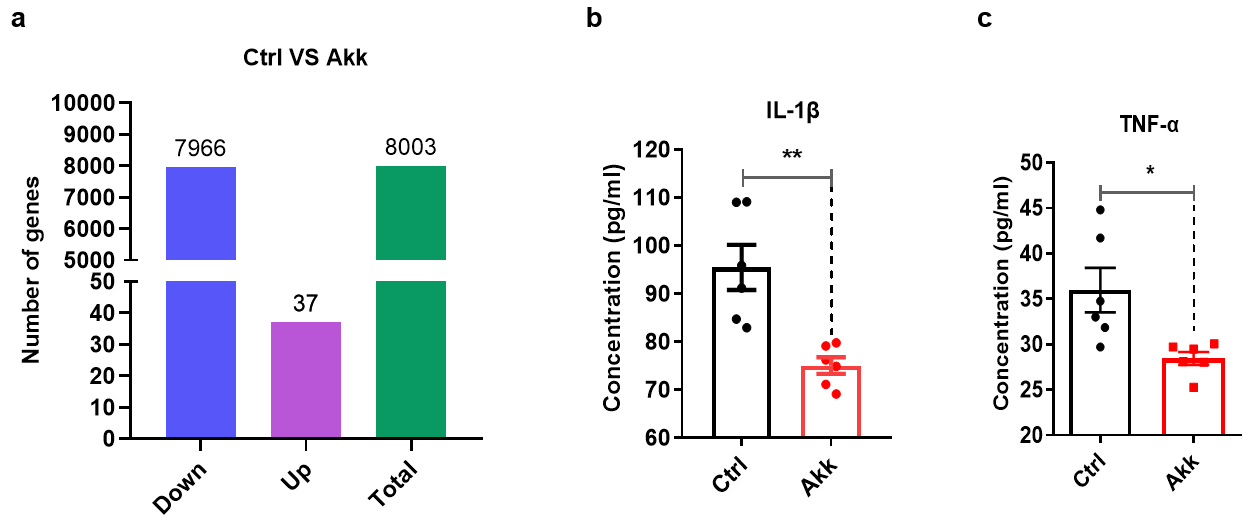


**Fig S4. *A. muciniphila* downregulated inflammatory-related gene and protein expression in the blood.** (a) The number of DGEs in the blood from aged mice treated with *A. muciniphila* compared with that in the control group. (b, c) An ELISA confirmed that the key proinflammatory cytokines IL-1β and TNF-α were significantly decreased after *A. muciniphila* treatment. Ctrl: Control; AKK: *A. muciniphila*. The overall significance between two groups was determined by Student’s *t*-test. * *p* < 0.05, ** *p* < 0.01, *** *p* < 0.001, ns, not significant.


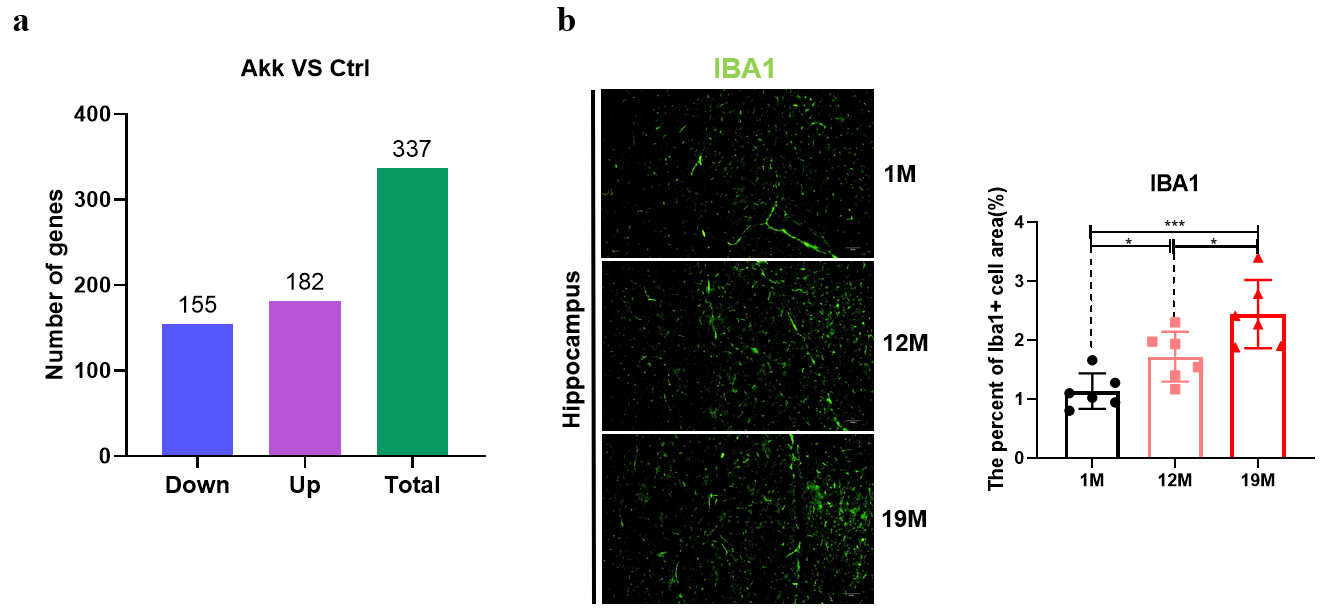


**Fig S5. The number of microglia gradually increased with age.** (a) The number of DGEs in the hippocampus of aged mice treated with *A. muciniphila* compared with that in the control group. (b) The number of microglia in the hippocampus areas gradually increased with age. Ctrl: Control; AKK: *A. muciniphila*. The overall significance among three or four groups was determined by one-way-ANOVA. * *p* < 0.05, ** *p* < 0.01, *** *p* < 0.001, ns, not significant.


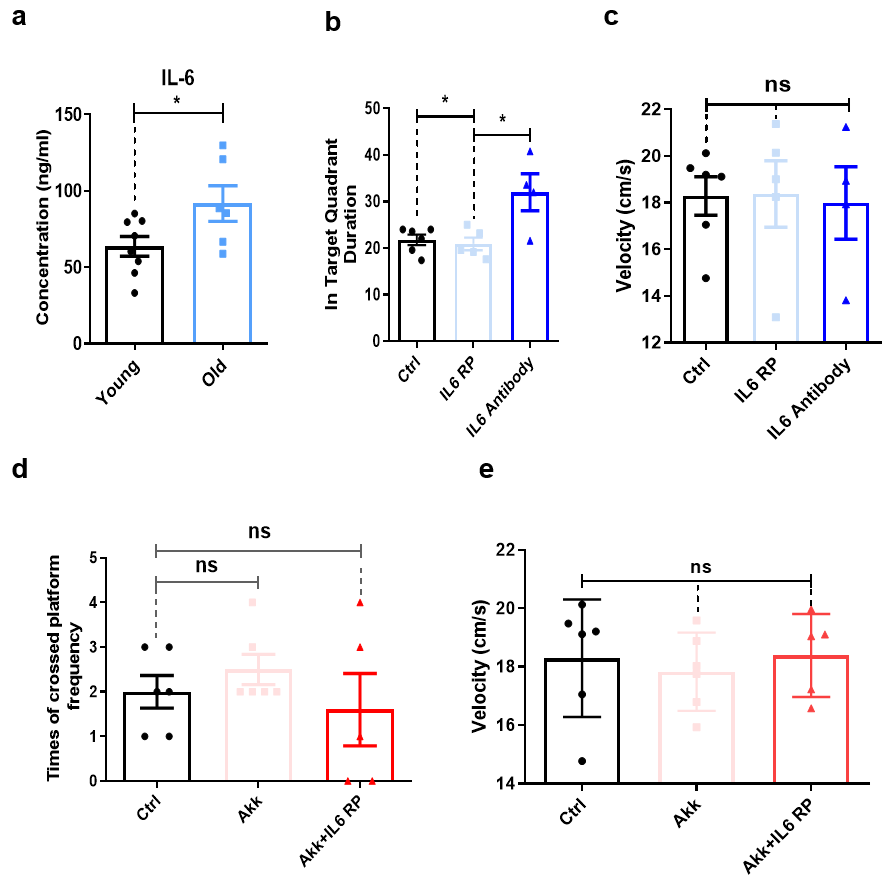


**Fig S6. The MWM was used to detect cognitive function changes after IL-6 and *A. muciniphila* treatment.** (a) The plasma level of the cytokine IL-6 was increased in aged mice. (b) Probe tests conducted after the acquisition phase indicated that IL-6 antibody-treated mice spent significantly more time in the target quadrant than control and IL-6 recombinant protein-treated mice. There was no difference between the IL-6 recombinant protein-treated and control groups during the probe test. (c) The mean velocity was not different among the three groups. (d) The time in the target quadrant was slightly increased in the *A. muciniphila-*treated group compared with that in the control and *A. muciniphila*+IL-6 recombinant protein-treated groups. (e) The latency to first crossing the previous platform location was slightly decreased in the *A. muciniphila-*treated group compared with those in the control and *A. muciniphila*+IL-6 recombinant protein-treated groups. Ctrl: Control; AKK: *A. muciniphila*. The overall significance among three or four groups was determined by one-way-ANOVA. * *p* < 0.05, ** *p* < 0.01, *** *p* < 0.001, ns, not significant.
